# Unconscious processing under interocular suppression: getting the right measure

**DOI:** 10.3389/fpsyg.2014.00387

**Published:** 2014-05-06

**Authors:** Timo Stein, Philipp Sterzer

**Affiliations:** ^1^Center for Mind/Brain Sciences (CIMeC), University of TrentoRovereto, Italy; ^2^Department of Psychiatry, Charité Universitätsmedizin BerlinBerlin, Germany; ^3^Berlin School of Mind and Brain, Humboldt-Universität zu BerlinBerlin, Germany; ^4^Bernstein Center for Computational NeuroscienceBerlin, Germany

**Keywords:** unconscious processing, visual awareness, process dissociation, interocular suppression, continuous flash suppression

In order to demonstrate unconscious visual processing, researchers need to select a technique for rendering stimuli invisible and a measure reflecting the processing of these stimuli. The most popular techniques are backward masking, in which the visibility of a very brief stimulus is degraded by the presentation of a succeeding visual pattern (Breitmeyer and Öğmen, 2006), and interocular suppression, where a stimulus shown to one eye degrades the visibility of a stimulus presented to the other eye (Lin and He, 2009). Recently, much work has been carried out using continuous flash suppression (CFS; Tsuchiya and Koch, 2005), a particularly potent interocular suppression technique. In CFS, a train of high-contrast patterns flashed into one eye can suppress the visibility of a stationary stimulus shown to the other eye for up to several seconds (Figure 1). Because CFS allows for extended periods of reliable invisibility of complex stimuli, this technique has sparked a surge of interest in unconscious visual processing.

**Figure 1:**
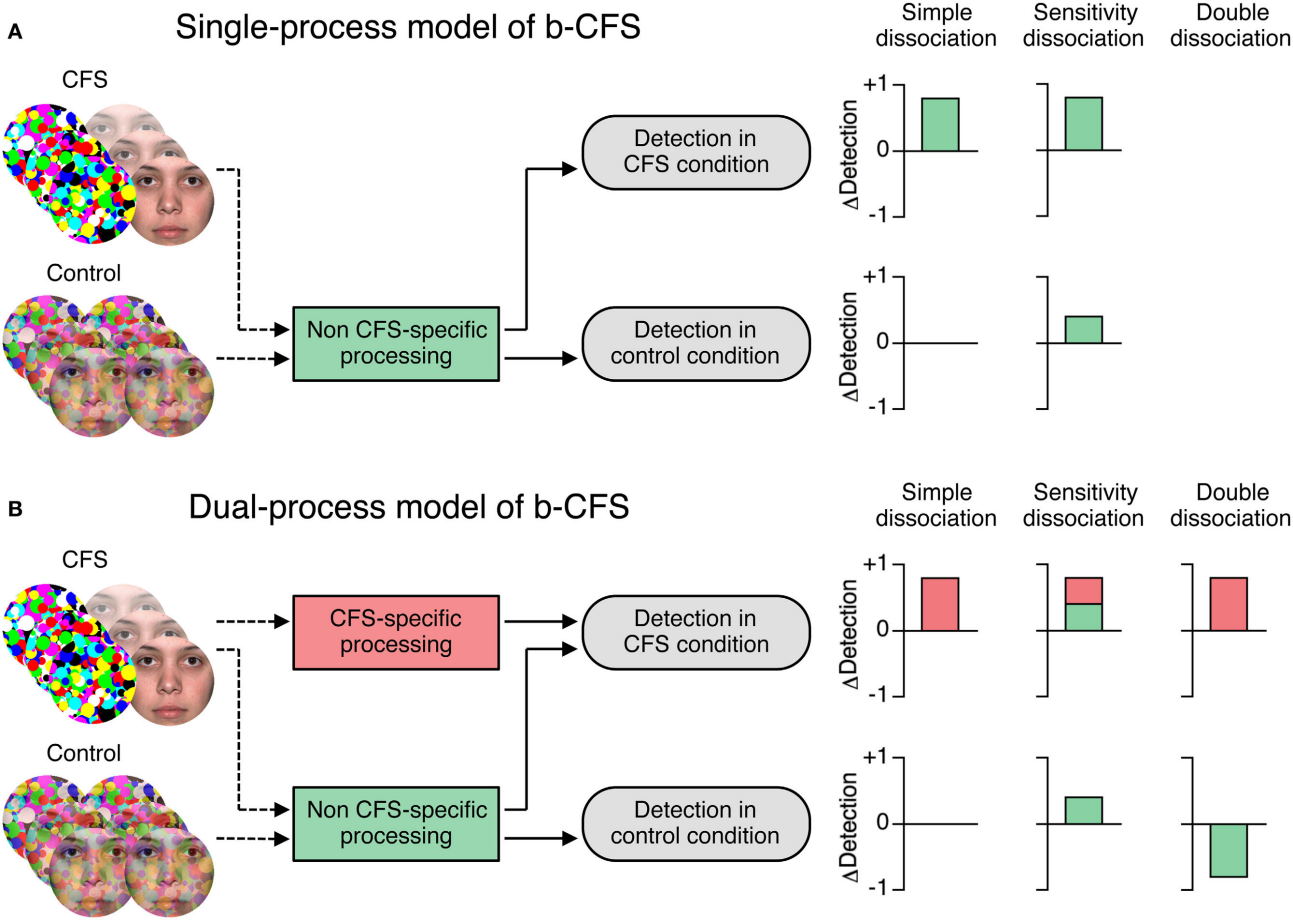
**Competing models of the processes mediating detection performance in the b-CFS paradigm. (A)** The single-process model posits that both the CFS and the control condition measure differences between stimuli in accessing awareness that exist independent of the application of CFS, i.e., that are caused by *non CFS-specific* processing differences (green boxes and bars). The bar graphs on the right show how the single-process model would account for hypothetical detection differences (ΔDetection in arbitrary units) between two stimuli in the CFS condition (top) and in the control condition (bottom). To account for simple and sensitivity dissociations, the single-process model would need to assume that the CFS condition represents a more sensitive measure of non CFS-specific processing differences than the control condition. The single-process model cannot account for double dissociations. **(B)** The dual-process model posits that differences in conscious detection between stimuli obtained in the CFS condition are at least partly due to the application of CFS, i.e., they reflect *CFS-specific* processing differences (red boxes and bars). In the b-CFS literature, simple and sensitivity dissociations between the CFS and the control condition have been take to support the dual-process model. The dual-process model assumes that CFS-specific processing accounts for effects that are larger in the CFS than in the control condition, as illustrated by the red bar graphs on the right. We, however, suggest that double dissociations between the two conditions (i.e., some experimental manipulation has opposite effects on detection in the CFS and the control condition) are required to refute the single-process model and to postulate distinct CFS-specific processing.

Ideally, research aimed at delineating the scope and limits of visual processing without awareness should adopt the technique that is most sensitive to unconscious processing. This is because a failure to find evidence for a certain unconscious effect could always be due to constraints imposed by the specific technique rather than to the genuine absence of unconscious processing (Faivre et al., 2012). However, since the extent to which a technique allows for unconscious processing is difficult to determine, and due to a lack of general consensus on valid measures of unconscious processing, no definite criteria exist for choosing the most sensitive technique.

## Classic dissociation approaches to unconscious processing

Most commonly, unconscious processing is studied using some variant of the classic dissociation paradigm, in which a direct measure of stimulus awareness (e.g., subjective ratings or objective discrimination performance) is contrasted with an indirect measure of stimulus processing (e.g., priming effect). For this comparison to be valid, both measures need to be obtained using identical stimuli and stimulus-response mappings, and the direct task needs to assess awareness of the critical stimulus manipulation that is driving the effect in the indirect measure (Schmidt and Vorberg, 2006). Thus, the only difference between the two measures should lie in the task instructions, with the direct task referring explicitly to the critical stimulus manipulation (Reingold and Merikle, 1988). Schmidt and Vorberg (2006) described three types of dissociations that can provide evidence for unconscious processing, depending on some critical assumptions: (1) The direct measure has null sensitivity while the indirect measure has some sensitivity. This *simple dissociation* requires the direct measure to capture all aspects of relevant conscious perception. (2) The indirect measure has greater sensitivity than the direct measure. This *sensitivity dissociation* requires the direct measure to be at least as sensitive to relevant conscious perception as the indirect measure. (3) Some manipulation has opposite effects on the indirect and the direct measure. Such *double dissociations* may provide the most compelling evidence for the existence of two distinct underlying processes (Mattler, 2003; Vorberg et al., 2003; Lau and Passingham, 2007).

The majority of studies adopting the classic dissociation paradigm followed the simple dissociation logic, probably due to its face validity and practical feasibility. This approach has provided clear evidence for high-level visual and semantic unconscious processing under backward masking (Kouider and Dehaene, 2007; Van den Bussche et al., 2009). Under interocular suppression, by contrast, unconscious processing seems to be comparably limited (Tong et al., 2006; Almeida et al., 2008; Lin and He, 2009). For example, a number of studies have failed to obtain evidence for unconscious processing of facial features rendered invisible through CFS (Moradi et al., 2005; Shin et al., 2009; Yang et al., 2010; Amihai et al., 2011; Stein and Sterzer, 2011; Stein et al., 2012a; but see Adams et al., 2010; Xu et al., 2011; Barbot and Kouider, 2012). This indicates that backward masking represents a more sensitive technique for measuring unconscious high-level processing than interocular suppression.

## Breaking continuous flash suppression (b-CFS)

This notion has recently been challenged by findings obtained with the novel breaking continuous flash suppression (b-CFS) paradigm in which differential unconscious processing during CFS is inferred from the time different stimuli need to overcome CFS and break into awareness, as reflected in speeded localization (or detection) responses (Jiang et al., 2007). A rapidly growing body of literature using b-CFS now suggests that interocular suppression allows for a much greater extent of high-level unconscious processing than previously thought (for a review, see Gayet et al., submitted). For example, b-CFS is sensitive to various features of face stimuli (Jiang et al., 2007; Yang et al., 2007; Zhou et al., 2010; Stein et al., 2011b,c, 2012b, 2014; Chen and Yeh, 2012; Stein and Sterzer, 2012; Stewart et al., 2012; Gobbini et al., 2013a,b), and can even be influenced by semantic stimulus properties (Costello et al., 2009; Mudrik et al., 2011; Sklar et al., 2012). These findings demonstrate that b-CFS is highly sensitive to differences between complex stimuli in their potency to gain access to awareness.

However, detection or localization responses as used in b-CFS represent a measure of conscious stimulus processing. In the classic dissociation paradigm b-CFS would thus count as a *direct* measure of stimulus awareness. Why then is b-CFS typically regarded as a measure of unconscious processing? One possibility is that, because target stimuli in b-CFS remain invisible for up to several seconds, differences in detection time may seem to suggest that the visual system discriminates between stimuli before conscious access, i.e., unconsciously. However, the same logic could in principle be applied to any visual detection measure (Gaillard et al., 2006). Consequently, findings from all experiments measuring visual detection, such as tasks designed as awareness checks, paradigms for measuring contrast detection thresholds, visual search, or attentional blink would need to be reinterpreted as evidence for unconscious processing. Clearly, this interpretation is in direct contradiction to a long history of research into unconscious processing that adopted the classic dissociation logic.

Alternatively, and more likely, is that unconscious processing is inferred from b-CFS only *because* CFS is used to degrade stimulus visibility. That is, differences in access to awareness are attributed to differential processing that occurred specifically under CFS, i.e., to *CFS-specific* processing differences. For this reasoning to be valid, *non CFS-specific* threshold differences need to be ruled out as a cause for differences in access to awareness. To isolate CFS-specific processing, most b-CFS studies contrasted detection performance under CFS with a binocular control condition. This control condition implements the same detection task as the CFS condition, but stimuli are presented binocularly, with the target stimulus gradually blended in on top of the flashing masks. The control condition is intended to capture all non CFS-specific processing differences that could play a role in the CFS condition.

## A process-dissociation framework for b-CFS

Thus, the b-CFS paradigm aims to show a dissociation between the CFS and the control condition in order to provide evidence that CFS-specific processing drives detection performance in the CFS condition. That is, b-CFS studies attempt to refute a single-process model in favor of a dual-process model. The single-process model posits that detection performance in both the CFS and the control condition reflects non CFS-specific processing (Figure 1A). By contrast, the dual-process model posits that detection performance in the CFS condition is at least partly mediated by CFS-specific processing (Figure 1B). This dissociation logic is markedly different from the classic dissociation paradigm described above, in that both the CFS and the control condition are direct measures that use different stimuli but identical tasks.

To date, b-CFS studies have inferred CFS-specific unconscious processing when an effect was found in the CFS condition but none in the control condition (*simple dissociation*) or when the effect in the CFS condition was larger than in the control condition (*sensitivity dissociation*). These dissociations require the control condition to be at least as sensitive as the CFS condition to all aspects of non CFS-specific processing that might have contributed to the effect in the CFS condition. We have recently shown that this critical assumption is unwarranted, because the CFS and the control condition are not directly comparable and differ in various aspects other than CFS-specific processing (Stein et al., 2011a). Thus, simple or sensitivity dissociations could be due to factors other than CFS-specific processing. In fact, it is possible that the CFS condition is simply a more sensitive measure of non CFS-specific differences in stimulus detectability than the control condition. Hence, simple and sensitivity dissociations cannot provide unequivocal evidence for CFS-specific processing.

However, a *double dissociation* between the CFS and the control condition could be used to directly refute the single-process model. The only assumption required is that non CFS-specific processing differences influence the CFS and the control condition in the same direction. If some experimental manipulation had opposite effects on detection in the CFS and the control condition, this would be inconsistent with the notion that non CFS-specific processing differences were driving the effect in both conditions. Therefore, a dual-process model would be required to fit the data. To illustrate, if an accuracy-based, criterion-free sensitivity measure revealed that under CFS neutral words were detected better than negative words (for response time based evidence, see Yang and Yeh, 2011), whereas in the control condition negative words were detected better than neutral words, this would establish a double dissociation.

Although double dissociations would provide convincing evidence that distinct processes mediate detection in the CFS and the control condition, opposite effects in the CFS and the control condition may be the exception rather than the rule and thus difficult to obtain in practice. Moreover, while double dissociations establish the dissociation of processes, the labels (“conscious” vs. “unconscious”) assigned to these processes need to be postulated a priori (Cardoso-Leite and Gorea, 2010). Evidence for a separate process governing detection under CFS would thus not necessarily imply that this process takes place unconsciously. We nevertheless believe that the demonstration of double dissociations is essential for proving the dual-process model of b-CFS and may thus represent the critical first step on the road to a new direct measure of unconscious processing.

## Objective vs. subjective measures in b-CFS

Another way of studying unconscious processing that is fundamentally different from the classic dissociation logic is to contrast a direct measure of objective discrimination performance with a direct measure of subjective awareness, such as confidence ratings. On this approach, unconscious processing is inferred when the subjective measure has null sensitivity while the objective measure has some sensitivity (blindsight-like *simple dissociation;* Kolb and Braun, 1995; Kunimoto et al., 2001), or when the objective measure has greater sensitivity than the subjective measure (*sensitivity dissociation;* Sandberg et al., 2011). Following this objective-subjective dissociation logic, future b-CFS studies could collect, on every trial, criterion-free measures of objective and subjective sensitivity rather than response time based detection measures. Dissociations between direct objective and subjective measures would demonstrate unconscious processing and could be compared to the magnitude of objective-subjective dissociations obtained with other psychophysical techniques, such as backward masking (Kanai et al., 2010).

With appropriate stimulus manipulations, objective-subjective dissociations in b-CFS could also be used to probe the extent of unconscious processing. For example, demonstrating greater sensitivity to neutral than negative words in the objective measure (cf. Yang and Yeh, 2011) while showing null sensitivity to both neutral and negative words in the subjective measure could be regarded as evidence for unconscious processing of word meaning. Although the objective-subjective dissociation logic for measuring unconscious processing is still under development and an agreement on a valid, bias-free measure of subjective awareness has yet to be reached (Evans and Azzopardi, 2007; Sandberg et al., 2010; Maniscalco and Lau, 2012; Barrett et al., 2013), we believe that this approach represents a promising future application for b-CFS.

## Conclusion

For the time being, b-CFS cannot provide evidence for unconscious processing. We therefore suggest that findings from b-CFS that were originally taken as evidence for the processing of “invisible” or “unconscious” stimuli need to be reinterpreted as evidence for differences in mere stimulus detectability. Only studies adopting the well-established classic dissociation paradigm can provide unequivocal evidence for unconscious processing and guide the choice of the most sensitive psychophysical technique for rendering stimuli invisible.

### Conflict of interest statement

The authors declare that the research was conducted in the absence of any commercial or financial relationships that could be construed as a potential conflict of interest.
